# Metabolic syndrome and its components in patients with psoriasis

**DOI:** 10.1186/2193-1801-3-612

**Published:** 2014-10-17

**Authors:** Mercè Albareda, Anna Ravella, Marta Castelló, Sandra Saborit, Laura Peramiquel, Lluís Vila

**Affiliations:** Endocrinology Department Hospital de Sant Joan Despí Moisés Broggi. CSI, Jacint Verdaguer st, 90, 08970 Sant Joan Despí, Spain; Dermatology Department Hospital Dos de Maig. CSI, Dos de Maig st, 301, 08025 Barcelona, Spain; Dermatology Department, Hospital de Sant Joan Despí Moisés Broggi. CSI, Jacint Verdaguer st, 90, 08970 Sant Joan Despí, Spain

## Abstract

Psoriasis is a chronic inflammatory disease of the skin which affects 1-3% of the population. A higher association of metabolic syndrome (MS) has been described amongst sufferers. The objective of this study was to assess the association of MS and its components amongst subjects suffering psoriasis and compare it with that found for the control group. The secondary objective was to study the relationship between the duration and severity of the psoriasis and the MS. This was a case–control study: 102 subjects with psoriasis and 102 control subjects paired by sex, age and body mass index. Anamnesis: history of diabetes mellitus, arterial hypertension, dyslipidaemia and psoriasis. Lifestyle. Physical examination: weight, height, blood pressure, waist circumference. Tests: lipid profile, oral glucose tolerance test and insulinemia (HOMA calculation). MS classified according to the 2009 consensus. The prevalence of MS amongst psoriasis patients was 52.9%, as compared to 34.31% in the control group. MS independent factors: age (OR 1.085), body mass index (OR 1.346), sex (OR 2.69 for men) and psoriasis (OR 3.634). A comparative study of patients with psoriasis with or without MS, revealed no relationship to the severity, age at time of diagnosis or time of evolution of the psoriasis. In conclusion, the association of MS amongst psoriasis sufferers is very high and the disease is considered as an independent risk factor for MS. Our results show no relationship between the different characteristics of psoriasis and the presence of MS. The main limitation of this study is that it does not enable to conclude whether psoriasis is a risk factor for MS or the opposite.

## Introduction

Psoriasis is a genetic multifactorial disease of the skin, which affects approximately 2% of the population. The cause of the disease remains unknown. It has been suggested that it is probably caused by some kind of autoimmune mechanism, though the triggering antigen has yet to be identified.

Over recent years, a series of publications has appeared showing an increased frequency of metabolic syndrome (MS) and its components amongst subjects with psoriasis (Sommer et al. 2006; Gisondi et al. 2007; Neimann et al. 2006; Prey et al. 2010; Armstrong et al. 2013), leading in turn to an increased risk of cardiovascular disease and death (Sommer et al. 2006; Ludwig et al. 2007). Said association would not appear to be related to age, sex or the kind of psoriasis (Sommer et al. 2006; Gisondi et al. 2007), but there is some dispute with regard to its relationship to the severity and duration of the disease (Gisondi et al. 2007; Prey et al. 2010).

Amongst these patients, a higher level of prevalence of a series of factors has also been described, which could account for the more extensive presence of MS and heightened cardiovascular risk. Such factors include tobacco addiction, obesity, physical inactivity, depression, poor food habits and psychological stress (Sommer et al. 2006; Sterry et al. 2007; Christophers 2006; Hamminga et al. 2006).

The etiopathogenetic relationship between the two processes is not entirely known. However, the presence of certain pro-inflammatory cytokines and immunological mediators has been identified in both diseases (Sterry et al. 2007). Some authors suggest that the heightened incidence of MS in psoriasis sufferers could partly be explained by the chronic presence of systemic inflammation with psoriasis (Prey et al. 2010; Shapiro et al. 2012). However, in MS a proinflammatory state is also to be found and certain studies indicate that it is obesity which predisposes the body to developing psoriasis (Setty et al. 2007).

The aim of our study is to assess the association of MS and its components in a sample of subjects with psoriasis and to compare it to that of a control group of the same age, sex and body mass index (BMI). The secondary object is to study the relationship between the duration and severity of the psoriasis and the MS.

## Methods

### Study protocol

The present is a case–control study. It was approved by the research committee of Consorci Sanitari Integral and it was performed during the course of Dermatology consultations at the Barcelona Hospital Dos de Maig (level 2 hospital). Included in the study are patients with psoriasis who attended the hospital regularly and signed the informed consent form. The control group was recruited from amongst hospital workers or family members with no history if psoriasis. Control subjects were individually paired with psoriasis subjects according to sex, age and BMI.

The size of the sample was calculated with a view to the main object of the study, that is, to assess the prevalence of MS amongst a sample of subjects with psoriasis and compare it to that of a control group. To do so, we based our calculations on the (Love et al. 2011) study, which described a percentage of MS amongst the population with psoriasis of 39.9%, as compared to the 23.5% of the control population (a difference of 16.4%). To determine sample size for the present study, we assumed an expected level of prevalence amongst subjects with psoriasis of 30%. Given an α risk of 0.05 and a β risk of 20% in bilateral contrast, we required 97 subjects for each group to enable us to detect a difference of 16.4% in the prevalence of MS as a statistically significant difference between the two groups.

All psoriasis patients attending the Dermatology Department were asked if they would like to participate in the study. If the subjects accepted, the following protocol would then be followed: 1) Anamnesis: family and personal history of any of the components of MS or cardiovascular disease as well as any other pathological, pharmacological, diet related, tobacco and/or alcohol addiction (<3 vs. ≥3 alcoholic drinks per day), exercise (light, moderate or high) history, collected by way of the IPAQ (short version) questionnaire and the history of the psoriasis (onset, evolution, type, previous and current treatment, presence of psoriatic arthritis). 2) Physical examination: weight, height (calculation of BMI), waist circumference, systolic and diastolic blood pressure (BP) (two readings in a seated position, taken five minutes apart with an automatic electronic OMRON device). Calculation of the Psoriasis Area and Severity Index (PASI) (Fredriksson and Pettersson 1978). 3) Analytical study: Haemogram, basal glycaemia, creatinine, urea, GOT, GPT, lipid profile total cholesterol (TC), HDL-cholesterol (HDLc), LDL-cholesterol (LDLc), triglycerides (TG), TSH, free T4 and basal insulinemia. 4) Oral Glucose Tolerance Test with 75 g (glycaemia 120 minutes). 5) Estimation of insulin sensitivity by the HOMA method. The control subjects, after signing the informed consent form, followed the same protocol, with the exception of any assessment of the extent of psoriasis.

The presence of MS was assessed in line with the 2009 consensus criteria (Alberti et al. 2009): MS is considered present when subjects present 3 or more of the following: Waist circumference ≥80 cm for women and ≥94 cm for men (all subjects European); triglycerides ≥150 mg/dL (1.7 mmol/L) or hypolipemiant treatment; HDL <50 mg/dL (1.3 mmol/L) for women and <40 mg/dL (1,0 mmol/L) for men; BP ≥130/85 or hypertension (HT) treatment; basal glycaemia ≥100 mg/dL (or hypoglycaemiant treatment, or glucose intolerance or OGTT or diagnosed diabetes mellitus (DM)).

### Analytical studies

Analytical samples were taken after 12 hours of night fasting, and 120 minutes after the administration of 75 g of oral glucose. The methods used to determine the different parameters were as follows: Glucose: Hexokinase, Dimension®; CT: molecular absorption spectroscopy, cholesterol esterase and oxidase enzyme method, Dimension®; direct LDLc: spectro-enzymatic assay with cholesterol esterase and oxidase. Dimension®; HDLc: spectro-enzymatic assay with cholesterol esterase and oxidase. Dimension®; TG: molecular absorption spectroscopy, enzyme assay with lipoprotein lipase, Dimension®; Insulinemia: chemiluminescent microparticle immunoassay (CMIA), Architect®.

### Statistical analysis

We started with a descriptive analysis of the data. For qualitative variables, percentages and the corresponding 95% confidence interval were applied, while for quantitative variables, average and standard or mean deviation and minimum and maximum values were all employed.

The comparison of the groups with/without psoriasis was performed by way of a bivariate analysis, taking the *χ*^2^ test for qualitative variables and Student’s *t*-test for quantitative variables. If the latter were found not to comply with the normality assumptions, the Mann–Whitney *U* test was used.

Lastly we performed a logistic regression analysis, taking MS and the components thereof as our dependent variables, and the diagnosis of psoriasis, sex, age, diet, exercise and tobacco addiction as independent variables. A further logistic regression analysis was performed on the subjects with psoriasis, taking MS as our dependent variable while our independent variables were both those found significant in the bivariate study and others found clinically relevant. A second analysis was then carried out, differentiating between mild and moderate-severe psoriasis (mild PASI ≤10 with no systemic treatment; moderate: PASI >10 and/or systemic treatment: oral corticosteroids, methotrexate, biological treatment, retinoids).

For all the above tests, statistical significance was set at a level of 5%.

All statistical analysis was performed on an IBM SPSS v-19 statistical software package.

## Results

204 patients participated in the study, 102 affected by psoriasis and 102 with no history of psoriasis (55 men and 47 women in each group). Average age was 49.32 ± 13.47 years and average BMI index 27.7 kg/m^2^ (18.9-41.79) for the group with psoriasis, while 48.71 ± 13.84 years and 27.36 kg/m^2^ (18.24-40.5) were the averages found for the control group. Table 1 shows a description of the study population.

**Table 1:** **Description of subjects with/without psoriasis studied**

Characteristics	Subjects with psoriasis	Subjects free from psoriasis	***p-values***
	Mean ± SD	Mean ± SD	
	Median (minimal-maximal values)	Median (minimal-maximal values)	
Sex (♂/♀)	55/47	55/47	ns
Age (years)	49.32 ± 13.47	48.71 ± 13.84	ns
BMI (kg/m^2^)	27.7 (18.9-41.79)	27.36 (18.24-40.5)	ns
History			
- DM	14/102 (13.72%)	4/102 (3.92%)	0.024
- HT	27/102 (26.47%)	21/102 (20.58%)	ns
- DLP	36/102 (35.29%)	28/102 (27.45%)	ns
- CD	6/102 (5.9%)	4/102 (3.92%)	ns
Blood pressure(mmHg)			
- Systolic	130.75 (96-200)	124.5 (90-184)	0.006
- Diastolic	80.67 ± 12.38	78.06 ± 10.72	ns
Waist circumference (cm)			ns
♂	99 (75-122)	98 (67-130)	
♀	87 (63-120)	83 (63-116)	
Active tobacco consumption	35/102 (34.31%)	23/102 (22.54%)	0.063
Dietary control	24/102 (23.52%)	15/102 (14.7%)	ns
Exercise			
- Low	16/102 (15.6%)	17/102 (16.6%)	ns
- Moderate	47/102 (46%)	59/102 (55.8%)	
- High	35/102 (34.3%)	23/102 (22.5%)	
Consumption of alcohol (≥3/day)	55/102 (53.9%)	67/102 (65.6%)	ns
Fasting glycaemia (mg/dL)	99.1 (68.5-232.5)	91.95 (68.5-151)	ns
Glycaemia 120 min (mg/dL)	93.7 (45.1-264.9)	95.5 (48.7-286)	ns
HOMA Index	2.66 (0.85-22.73)	2.28 (0.51-9.42)	<0.001
Total Cholesterol (mg/dL)	201.16 (116.5-286)	197.17 (127-301.5)	ns
HDL cholesterol (mg/dL)	50.04 ± 11.91	51.66 ± 13.74)	ns
LDL cholesterol (mg/dL)	124.56 ± 28.77	128.02 ± 29.34	ns
Triglycerides (mg/dL)	98.19 (27.13-787.5)	87 (28.9-540)	ns

BMI: Body Mass Index. DM: Diabetes Mellitus. DLP: Dyslipidaemia. HT: Arterial Hypertension. CD: Coronary disease.

Fifty-three subjects (52.9% CI 95%: 42.4-61.4) with psoriasis presented with MS and 35 (34.31% CI 95% 25.8-43.9) from the control group (p = 0.016). With respect to the diverse components of MS, no significant difference was observed, even though there was a tendency towards a higher frequency of dyslipidaemia (DLP) amongst psoriasis sufferers (Table 2).

**Table 2:** **Prevalence of MS and the components thereof in the subjects studied with/without psoriasis**

	Subjects with psoriasis	Subjects without psoriasis	***p-values***
DM History			
Fasting Gly 100–126 mg/dL	51 (50%)	39 (38.23%)	ns
Gly 120 min 140–199 mg/dL			
HT			
BP ≥130/80	58 (56.8%)	48 (47.05%)	ns
DLP			
HDL <50 mg/dL ♀			
<40 mg/dL ♂	60 (58.9%)	46 (45.01%)	P = 0.068
TG >150 mg/dL			
Waist circumference			
≥80 cm ♀	70 (68.6%)	66 (64.7%)	ns
≥94 cm ♂			
MS (≥3 components)	53 (51.9%)	35 (34.31%)	0.016

DM: Diabetes Mellitus. Gly: Glycaemia. HT: Arterial Hypertension. BP: Blood Pressure. DLP: Dyslipidaemia. HDLc: Cholesterol HDL. TG: Triglycerides. MS: Metabolic Syndrome.

A univariate analytical study, comparing the two populations, with and without psoriasis, showed higher systolic BP and greater insulin resistance amongst subjects with psoriasis. Moreover, the same subjects presented known DM with greater frequency (13.7 vs 3.7%) and had a greater tendency to active tobacco addiction.

The multivariate study, comparing the psoriasis and control populations, underscores an independent relationship between age (OR 1.085 CI 95% 1.049-1.123), BMI (OR 1.346 CI 95% 1.228-1.499), sex (OR 2.690 CI 95% 1.195-6.057 men) and psoriasis (OR 3.634, CI 95% 1.645-8.025), with respect to the development of MS (p < 0.001). An analysis of each of the different components is shown in Table 3.

**Table 3:** **Results of the multivariate study performed, taking each of the components of MS as a dependent variables and age, sex, diet, exercise, tobacco addiction, consumption of alcohol and history of psoriasis as independent variables**

	Odds ratio	Confidence interval 95%
**Metabolic Syndrome**		
Psoriasis	3.634	1.645-8.025
Age	1.085	1.049-1.123
Sex (♂)	2.690	1.195-6.057
BMI	1.356	1.228-1.499
**Impaired glucose tolerance**		
Psoriasis	2.179	1.075-4.416
Age	1.089	1.055-1.124
Sex (♂)	2.297	1.102-4.787
BMI	1.206	1.112-1.307
**HT or BP >130/85**		
Age	1.066	1.034-1.099
Sex (♂)	2.213	1.055-4.643
BMI	1.329	1.207-1.464
**Dyslipidaemia**		
Psoriasis	2.000	1.120-3.570
BMI	1.084	1.019-1.154
**Waist circumference >80 cm for ♀ or >94 cm for ♂**		
Age	1.080	1.038-1.125
Sex (♂)	0.127	0.042-0.387
BMI	1.992	1.601-2.477
Tobacco consumption	0.364	0.132-1.00

BMI: body mass index.

With respect to the characteristics of the psoriasis suffered by the subjects, 90 presented plaque psoriasis, 6 guttate, 4 palmoplantar, 1 localised pustular and 1 inverse psoriasis. The average age at the time of diagnosis of the disease was 29.46 ± 15.67 years while duration was 19 years (0.5-55). The PASI score was 6.4 (0–36.6). With respect to treatment, 4 followed no course of treatment, 35 one single form of treatment and the remainder received combined treatment. The treatment employed was as follows: 16 on PUVA, 34 on UVB B-E, 6 on immunosuppressors (4 on methotrexate,1 on leflunamide and 1 on methotrexate + deflazacort), 2 on biological treatment with adalimumab, 6 on retinoids, 66 on topical corticosteroids, 39 on calcipotriol, 10 on topical keratolytics, 3 on tazarotene, 1 on coal tar and 1 on topical tacrolimus. Twenty-one subjects (20.58%) mentioned they also presented psoriasic arthritis. A univariate study, comparing subjects with psoriasis alone with those with MS is shown as Table 4. The patients with psoriasis and MS were older, with a higher BMI, greater insulin resistance and were older at the time of the initial diagnosis of psoriasis. No relationship was observed with either the PASI score or when the subjects were classified as having mild or moderate-severe psoriasis.

**Table 4:** **Differences between subjects with psoriasis with or without MS**

	MS	No MS	***P***
	Mean ± SD	Mean ± SD	
	Median (minimal-maximal values)	Median (minimal-maximal values)	
Age (years)	55.45 ± 11.28	42.69 ± 13.16	<0.001
BMI (kg/m^2^)	30.5 (22.5-41.79)	25.52 (18.9-36.36)	<0.001
HOMA Index	3.79 (1.07-22.73)	1.94 (0.85-6.97)	<0.001
Age at time of diagnosis (years)	34.25 ± 16.29	24.6 ± 13.5	0.002
Time of evolution (years)	20 (0.5-55)	15 (1-51)	ns
PASI Index	6.95 (0-35)	4.8 (0-36.3)	ns
Moderate - Severe Psoriasis	24/53 (45.2%)	18/48 (36.7%)	ns
Psoriatic Arthritis (%)	22.64%	18.3%	ns
Topical corticosteroids	54.71%	71.4%	
Immunosupressors	4 methotrexate	1 leflunamide	
	1 methotrexate + deflazacort		
Biolological treatment	-	2 adalimumab	

BMI: body mass index. PASI Psoriasis Area and Severity Index.

The multivariate study comparing subjects with psoriasis, grouped according to whether or not they also presented MS, showed an age (OR 1.083 CI 95% 1.035-1.134) and BMI (OR 1.346 CI 95% 1.168-1.550) independent relationship. Neither the age at the time of diagnosis, the time of development of the disease or the PASI score served as independent markers for MS.

## Discussion

Several studies have been published recently showing a higher prevalence of MS and the components thereof amongst subjects suffering psoriasis, with MS figures varying between 4.3 and 40%, clearly higher than those found in the control populations (Sommer et al. 2006; Gisondi et al. 2007; Armstrong et al. 2013; Love et al. 2011; Nisa and Qazi 2010; Kutlu et al. 2011) and lower than the prevalence found in our sample. A recent systematic and meta-analytical review of the more observational studies describes an OR of 2.26 for MS in subjects with psoriasis (Armstrong et al. 2013). Certain variables, that could derive in differences between the studies, should be examined. Firstly, the criteria by which MS was assessed, which varied from study to study (WHO, NCEP, ATP III). Secondly, the population studied, with observations of the Spanish population (VIVA Study) revealing lower levels of MS when compared to the other European and American populations (Gabriel et al. 2009). Amongst the Spanish population, looking at non-diabetic subjects aged between 30 and 65 years and following NCEP ATP III criteria, an MS prevalence of 15% has been described (19.5% for men and 14.7% for women) (Gabriel et al. 2009). A more recent Spanish study (DARIOS Study), performed in 24670 subjects aged 35 to 74 years, the level of prevalence observed was 31% (Fernández-Bergés et al. 2012). Thirdly, the severity of the psoriasis has only been observed to be related to the presence of MS in certain studies (Prey et al. 2010; Langan et al. 2012). Finally, the prevalence of obesity: obese subjects face a greater risk of presenting different components of MS, according to most studies. Subjects with psoriasis presented obesity with greater frequency than the control group (Sommer et al. 2006; Gisondi et al. 2007; Love et al. 2011) and a systematic revision describes an OR of 1.18-5.49 for obesity in subjects suffering psoriasis (Prey et al. 2010).

In our study, we applied the MS criteria of the 2009 consensus (Alberti et al. 2009), which are stricter, and thus selected a greater number of subjects affected from both groups in comparison with VIVA study (Gabriel et al. 2009), but the prevalence of control group was similar to DARIOS Study (Fernández-Bergés et al. 2012). On the other hand, the prevalence of obesity was found to be 32.35%, somewhat higher than in the VIVA study, which placed it at 27% (Gabriel et al. 2009). This is accounted for by the fact that cases and controls were paired by BMI, thus meaning the case subjects were at greater risk of being overweight and obese.

With respect to the other MS risk factors, the relationship between MS and age and BMI is known and the higher risk of MS amongst men was also described for the Spanish population by the VIVA study (Gabriel et al. 2009).

As opposed to previous studies (Sommer et al. 2006; Neimann et al. 2006), we did not find MS components to be present more frequently amongst subjects with psoriasis, though we did observe a higher percentage of known DM, a tendency to more frequently encounter lipid alterations and higher average systolic blood pressure. These results concur with those of the Prey et al. review (Prey et al. 2010), in which no conclusive figures were seen with respect to HT, la DM and DLP. The differences encountered with respect to other studies could be accounted for by the prevalence of obesity in the control group which, in our study, was similar to that of the psoriasis group, while in other studies it was lower, possibly thus creating more differences between the cases and controls. On assessing the components of MS, psoriasis continues to stand as an independent risk factor with respect to altering levels of glucose tolerance and dyslipidaemia. Subjects with psoriasis have a higher percentage of known DM, but no difference in the percentage of patients with impaired glucose tolerance between groups was observed. Patients with psoriasis have also an increased insulin resistance and probably worse impaired glucose tolerance, therefore a higher risk of diabetes.

On the other hand, we must point out that the subjects with psoriasis also presented greater insulin resistance, thus supporting the findings of the earlier study by Ucak et al. (Ucak et al. 2006). Additionally, an improvement to psoriasis has been described when under treatment with pioglitazone, a drug which enhances insulin sensitivity (Shafiq et al. 2005) and when there weight-loss improves the response to treatment with cyclosporine for subjects with moderate-severe psoriasis (Gisondi et al. 2008).

Despite this relationship between insulin resistance and the way the psoriasis responds to treatment, there are controversial results concerning the severity and duration of the disease and the MS (Sommer et al. 2006; Gisondi et al. 2007; Prey et al. 2010; Love et al. 2011; Nisa and Qazi 2010; Langan et al. 2012; Ucak et al. 2006). Neimann et al. (Neimann et al. 2006) describe a greater frequency of cardiovascular risk factors, both with mild and severe psoriasis, and associate severe psoriasis with a higher percentage of DM. Other studies find no relationship with severity, but do find a relationship to a greater duration of the psoriasis or younger age at diagnosis (Gisondi et al. 2007; Nisa and Qazi 2010). Sommer et al. (Sommer et al. 2006) describe a greater risk of MS in subjects hospitalised with severe psoriasis while other studies performed with ambulatory subjects with less severe disease rule out any such relationship (Gisondi et al. 2007; Kutlu et al. 2011; Ucak et al. 2006), as does our study. This could point to it being related to severity, given that it was found in more severely affected patients and not those with milder forms of the disease or to, as suggested by Gisondi et al. (Gisondi et al. 2007), the fact that a want of any kind of relationship would tend to indicate that it was the obesity itself that was favoured by the psoriasis. Finally, a more recent study described a clear relationship between MS and the severity of psoriasis, with 14% of MS found in subjects with mild psoriasis, 34% with moderate and 66% with severe disease. The results offered by the Setty et al. (Setty et al. 2007) study support this hypothesis, and relate BMI and weight increase with the diagnosis of new cases of psoriasis (Setty et al. 2007). On the other hand, it should be noted that several different ways of assessing the severity of psoriasis were employed by the different studies: the PASI Index (Sommer et al. 2006; Kutlu et al. 2011), the form of treatment the patient was on (Neimann et al. 2006) and the percentage of body surface affected (Langan et al. 2012). Our study used the PASI Index and treatment, but quite probably the fact that the patients were not freshly diagnosed but were mostly already on a course of treatment, could mean that the PASI Index would indicate the severity of the disease at the moment of the study, but not of its earlier severity during the course of the subject’s history with the disease.

Our study presents certain limitations. Firstly, since it is a cross-sectional study, it does not enable one to observe the onset and evolution of the relationship between psoriasis and MS. Secondly, and as already mentioned above, the fact that the patients were already on treatment could affect assessment of the severity of the psoriasis with the PASI Index and could also modify the components of MS, as would be the case for treatment with oral corticosteroids, cyclosporine, methotrexate or biological agents. In our study, there were two subjects under treatment with adalimumab (TNFα antagonist). There are studies which suggest that TNFα antagonists could have a beneficial effect on cardiovascular risk factors (Channual et al. 2009). A review of the effects of adalimumab treatment of MS for subjects with psoriasis, shows one case of hyperglycaemia in a subject with type 2 DM, while another study showed weight increase and an absence of lipid profile changes amongst 30 subjects (Channual et al. 2009). However, other studies performed with TNFα antagonists on subjects with rheumatoid arthritis and ankylosing spondylitis reveal different results (improvement and no change), but not an increase in insulin resistance (Ferraz-Amaro et al. 2011; Kiortsis et al. 2005). With our current results we are unable to either rule out or confirm any effect adalimumab may have on the MS of the two patients assessed, given that they were both found free of MS. With regard to methotrexate, diverse studies refer to it affording a reduction of cardiovascular risk (Micha et al. 2011) and thus in our study its possible effect on MS would be to reduce risk and thus lessen the differences between the two groups. Finally, we wish to underscore the possible skew there could have been in the choice of the control sample (health care deliverers and their families), chosen given difficulty of finding control subjects that could be paired with the subjects suffering psoriasis from amongst the psoriasis free patients visiting the Dermatology Department. It could be considered that health care deliverers have a healthier lifestyle, but any such factor will be eliminated when the subjects are compared by diet and also because of the level of prevalence of MS, clearly higher than in other studies carried out in our local environment due to the higher BMI of our subjects.

In summary, the present study also confirms a high percentage of MS amongst subjects with psoriasis and considers it an independent risk factor for MS, showing significantly higher levels than amongst the subjects from our control population and far higher than those found by other local studies. Consequently, it is important to detect it early for this group of patients, to be able to start treatment early and thus reduce the risk of cardiovascular disease. The present study is unable to confirm any relationship between the characteristics of psoriasis and MS which could indicate that subjects with psoriasis present a greater risk of MS.
